# Aspects of Elite Female Football Players’ Training Loads and Sleep Variations

**DOI:** 10.3390/sports12060163

**Published:** 2024-06-13

**Authors:** Kine Gjertsås, Frode Moen, Svein Arne Pettersen

**Affiliations:** 1 Department of Circulation and Medical Imaging, Faculty of Medicine and Health Sciences, Norwegian University of Science and Technology, 7030 Trondheim, Norway; 2 Department of Education and Lifelong Learning, Faculty of Social and Educational Sciences, Norwegian University of Science and Technology, 7030 Trondheim, Norway; frode.moen@ntnu.no; 3 School of Sport Sciences, Faculty of Health Sciences, UiT The Arctic University of Norway, 9010 Tromsø, Norway; svein.arne.pettersen@uit.no

**Keywords:** sleep, football, training load, recovery, performance

## Abstract

The current study investigated the associations between female football players’ training loads and their sleep variations. The sample included 21 female elite football players from a Norwegian top-league club with a mean age of 24 years (±2.8). Sleep duration, sleep quality, and training load were monitored every day over 273 consecutive days with a Somnofy sleep monitor based on ultra-wideband (IR-UWB) pulse radar and Doppler technology, and a FIFA-approved STATSports APEX 10 Hz GPS tracking system monitoring players’ training loads. A multivariate analysis of variance (MANOVA) was conducted to investigate the relationships between the players’ training loads and sleep. It was revealed that very high training loads were associated with reduced time in bed (*p* = 0.005), total sleep time (*p* = 0.044)), and rapid eye movement (*p* < 0.001). The present findings show that the female football players’ sleep was disrupted when the training load, based on total distance (TDI), was very high. It appears to be a point where their sleep is somewhat consistent through low, medium, and high training loads, but with disrupted sleep when the training load reaches a very high level. Considering the reduced TIB after a very high training load, there should be suggested strategies to improve their sleep, such as extended TIB, to aid in longer TST and improved recovery.

## 1. Introduction

Football involves a multitude of physical demands such as sprinting, rapid changes in direction, high running speeds, accelerations, decelerations, jumps, tackles, and various technical and tactical elements. [1]. These demands, along with the ever-changing nature of the game, require significant perceptual skills, making football a sport that is both physically and mentally demanding [2]. The physical exertion during matches or intense training sessions often leads to muscular fatigue and micro ruptures in muscle fibers, triggering an inflammatory response. This fatigue can stem from both central and peripheral mechanisms, with central fatigue affecting sprinting ability and maximal voluntary contraction, whereas peripheral fatigue is associated with muscle soreness and damage [3]. Muscle damage and inflammation contribute to decreased physical performance, requiring up to 72 h for full recovery post-match [1,4]. Additionally, there is considerable variability among players in terms of recovery needs, influenced by factors like match outcomes and positional differences. Female football players, for example, exhibit distinct physical demands across different positions, with central defenders typically experiencing less high-intensity activity compared to other positions [2].

To optimize performance and prevent injuries, players must carefully balance physical exertion with adequate recovery strategies, particularly in the face of multiple competitions within a short timeframe [4]. Sleep emerges as a crucial factor in this balance, serving essential psychological and physiological functions [5,6]. Despite its importance, elite football players often struggle to obtain sufficient and good-quality sleep due to various factors such as late kick-off times, caffeine consumption, and irregular sleep schedules [7,8,9].

Lately, in line with rapid technology development, more user-friendly, accurate, and non-intrusive radar technology for sleep assessment has been developed for use in research and hospital settings. Recent advancements in sleep monitoring have shed light on sleep patterns in athletic populations, revealing that a higher mental strain is associated with less TST (total sleep time), REM (rapid eye movement), and sleep efficiency (SE), and an increased training load is associated with a decrease in REM sleep in junior endurance athletes [10]. A study in elite endurance athletes found a decrease in TST and LS (light sleep), and an increase in DSL (deep sleep) and non-REM respiration rate (RPR) during a 3-week altitude training camp at 1800 m compared to baseline measurements near sea level [11]. Research on the impact of football on sleep patterns in female football players revealed a reduced sleep duration and later bedtime following matches compared to normal training days [12]. Sleep duration was lower the night after a match compared to the preceding day and match day [13]. Perceived fatigue was associated with increased time in bed (TIB) and deep sleep, and decreased perceived fatigue was related to an increase in REM sleep. This study also found that nights following football matches were associated with reduced TIB, TST, LS, DSL, and REM sleep, longer SOL (sleep onset latency), and an increased non-REM respiration rate [14]. A recent study also revealed that the players’ TST and LS, DSL, and REM sleep drop on match night, as the objective loads peak on match nights [7]. However, there remains a gap in understanding how different training loads affect sleep stages, particularly among female football players. Previous studies have largely relied on questionnaires or actigraphy, which provide limited insight into sleep stages.

The current study aims to investigate the relationships between objective training loads and sleep pattern in elite female football players. It hypothesizes that whereas increased training load may initially improve sleep (H1), exceptionally high loads could disrupt sleep patterns (H2). This research seeks to elucidate the intricate interplay between physical exertion, recovery, and sleep quality in elite football players, providing valuable insights for optimizing performance and well-being.

## 2. Materials and Methods

### 2.1. Participants

The current study is a part of data collection with several research questions [7]. Participants were recruited from a Norwegian elite female football team. Together with the coaching staff, all 25 players in the team were invited to an information meeting regarding the current research project. The purpose of the research project was explained in detail with a description of logistics, responsibilities for different tasks in the project, and the data collection process. Twenty-three football players participated in the current study and signed a written informed consent form approved by the local Regional Committee for Medical and Health Research Ethics (REC) in Central Norway (project ID 2017/2072/REK midt). One of the players withdrew from this study after a month of data collection and one person was omitted because of missing data. Thus, 21 female football players (mean age 23 ± 2.8, range 20–29 years) completed the current study.

### 2.2. Instruments

#### 2.2.1. Sleep

The Somnofy sleep monitor (version 0.7, VitalThings AS, Trondheim, Norway) is a unique, fully non-intrusive tool for sleep assessment, utilizing an impulse radio ultra-wideband (IR-UWB) pulse radar and Doppler technology. Somnofy is certified according to “Conformité Européene” (CE) and the Federal Communication Commission (FCC). The IR-UWB radar emits radio wave pulses in the electromagnetic spectrum, which can pass through soft materials (e.g., duvets or clothes), but is reflected by denser materials (e.g., a human body or a wall). The reflected pulses are then returned and received by the IR-UWB radar again. The time-of-flight is used to analyze the time it takes to cover the distance between the radar and the person and back to the radar again. The movements of the sleeping person and their respiration rate are derived from the IR-UWB radar by utilizing the Doppler effect and Fast Fourier Transform, allowing the Somnofy to monitor the movement and respiration of the individual with high precision. The raw data of movement and respiration are processed by a sleep algorithm, which uses machine learning to calculate relevant sleep variables. Somnofy is found to be a sufficient measure of sleep and wake cycles, and sleep stage distribution in healthy adults after a full validation study against the gold standard polysomnography (PSG) [14,15].

For the purposes of this research project, the following sleep variables were obtained from the Somnofy sleep monitor: Time in bed (TIB), sleep-onset latency (SOL), total sleep time (TST), time in light sleep (LS), time in deep sleep (DSL), time in REM sleep (REM), sleep efficiency (SE), and respiration rate (RPR). A description of these sleep variables is shown in Table 1.

#### 2.2.2. Training Load—GPS Tracking

To quantify the training loads and the movement patterns, the female football players were equipped with the FIFA-approved STATSports APEX (Statsports, Newry, Northern Ireland) system. Each player wore a GPS tracker on their upper back in a tight-fitted vest during both training and matches. Every player used the same GPS unit during the entire data collection to minimize inter-device errors. After every training session and match, the data from the units were retrieved and uploaded to the club’s laptop via the manufacturer’s software (STATSports Sonra 2.1.4). After the data collection was finished, the complete dataset was exported to the researchers. The raw data from each unit contain the ID of the football player, time, longitude, latitude, Doppler-derived speed (m/s), heart rate (bpm), horizontal accuracy (Hacc), horizontal dilution of precision (Hdop), quality of signal, and instantaneous acceleration impulse. All data were captured at 10 Hz, whereas Micro-Electro-Mechanical-System data were captured at 100 Hz. The validity of STATSports APEX shows good levels of accuracy (bias < 5%) in sport-specific metrics [16]. The GPS tracker obtained total sprint distance over 22.5 km/h, total distance, accelerations, decelerations, distance per minute, and players’ maximal sprint speeds. For the current study, total distance (TDI) was collected as a measure of the participants’ training loads. TDI is associated with other external loads such as high-intensity running, accelerations and decelerations, and internal indicators such as RPE (rating of perceived exertion) [17,18]. Thus, TDI is utilized as a measure of objective training load.

### 2.3. Procedure

All players were equipped with a Somnofy sleep monitor and given oral and written instructions on the correct use and placement of the sleep monitor for optimal functionality. The data collection entailed day-to-day monitoring of the football players’ sleep detected by the Somnofy sleep monitor and the on-field training loads collected by the STATSports APEX tracking system. The researcher maintained real-time access to a comprehensive overview of participant adherence within this study, diligently overseeing the progression to promptly identify and rectify any technical difficulties associated with the sleep monitoring and GPS tracking systems during the research period. This vigilant supervision facilitated the effective management of challenges inherent in the deployment of these technological systems, thereby guaranteeing the precision and dependability of data collection throughout the duration of this study. Data acquisition spanned both the pre-season and the official match season, extending from 1 February 2022 to 31 October 2022. Data collection activities were confined to the duration of the football season and did not extend beyond this period.

### 2.4. Statistical Analysis

IBM SPSS (version 27.0) was used to conduct descriptive statistical analyses, presented as mean ± standard deviation (S.D.). The outlier of sleep data defined as epoch counts of sleep sessions below 3 h (1000 s) and non-attendance sleep sessions greater than 3000 s were deleted. To avoid naps during the day and movements in the room being detected as sleep data, sleep sessions between 12.00 am and 20.00 pm were deleted. Data from GPS STATSports APEX tracking system less than 500 m in TDI were deleted to ensure no technical issues and extreme outliers. For each group of variables (TDI and sleep data), days without observations were omitted. This resulted in a total of 2033 observations of training data and sleep data, from each player each day.

**Figure 1:**
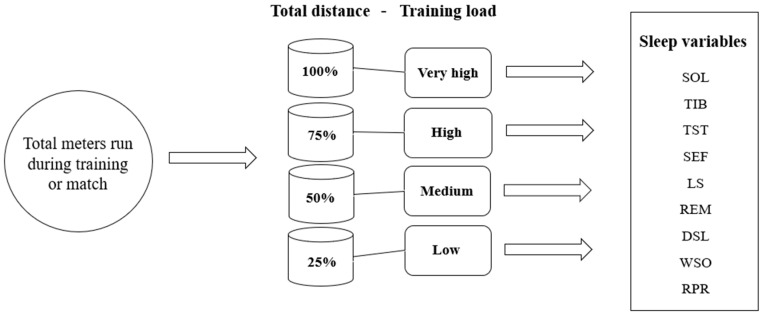
Associations between different training loads based on total distance and the sleep variables associated with the different classifications of loads.

TDI data obtained from on-field football practices and matches were classified into different training loads low, medium, high, or very high training loads. The classification of the training load was based on each player’s individual observations [2,19]. The classification of the training load was determined by percentiles (25, 50, 75, and 100). Each player’s dataset of training load based on TDI was divided into 100 equal parts and each part (percentile) represents a percentage of the data. The 25th percentile is categorized as a low training load, the 50th percentile is categorized as a medium training load, the 75th percentile is categorized as a hard training load, and the 100th percentile is categorized as a very hard training load. A low training load represents the 25% lowest observations in the dataset, a medium training load represents the values between the 25th and 50th percentiles in the observations, a high training load corresponds to the values observed between the 50th and 75th percentiles, and a very high training load corresponds to the 75th and 100th percentiles (Figure 1).

For the majority of the measures, a one-way repeated-measures Analysis of Variance was used to determine whether there were differences among the training loads. Where significant main effects were found, post hoc tests with Bonferroni correction were conducted to pinpoint the exact locations. For the measures that did not meet the statistical assumptions required for ANOVA, Kruskal–Wallis was used instead. The repeated-measures analysis was used because of the clustered data structure and to prevent excessive type 1 errors and biased parameter estimates.

Homogeneity of Variances was tested with Levene’s Test of Equality of Error Variances, and normal distribution with visual assessments of Q-Q plots and histograms were used to assess the normality of data to verify that the assumptions for MANOVA were met. Partial Eta Squared was used to test the effect size of the studied variables. The significance level was set to *p* < 0.05.

## 3. Results

The current study had a potential of collecting 6006 data points from sleep and physical load data using Somnofy sleep monitoring and GPS tracking over 273 consecutive days. Out of this, 4746 (79%) objective sleep data were collected and available for analysis. The missing data points were a result of inaccurate measurements, technical issues such as connecting Somnofy to Wi-Fi, travels where the football players forgot their sleep monitor, and when one player was transferred to another club in July. A total of 2497 (41.6%) data points of TDI were collected. The remaining data were lost due to training sessions where GPS tracking was not suitable to use, as in strength training, and to the football players’ forgetfulness. Overall, 2033 (33.8%) observations from the sleep data and training load data were investigated for 273 consecutive days. The omitted data were a result of non-matching days of data points from TDI and sleep variables, or GPS and sleep data indicating possible error.

Descriptive statistics (mean ± STD) of the studied sleep variables and TDI including all football players during the entire period of data collection are shown in Table 2. Of a total of 2033 observations, 501 data points were classified as a low training load, 510 data points as a medium training load, 517 data points as a high training load, and 505 data points as a very high training load.

Based on a series of Levene’s F tests, the homogeneity of variance assumptions was considered satisfied, although the Levene F tests of TIB (time in bed) and TST (total sleep time) were statistically significant (TIB = 0.026. TST = 0.009.) A Kruskal–Wallis Test of TIB and TST and total distance was then conducted as a non-parametric test equivalent to ANOVA. Kruskal–Wallis H = 13,138, *p* = 0.004 (TIB). Kruskal–Wallis H = 8.6697, *p* = 0.034 (TST).

**Figure 2:**
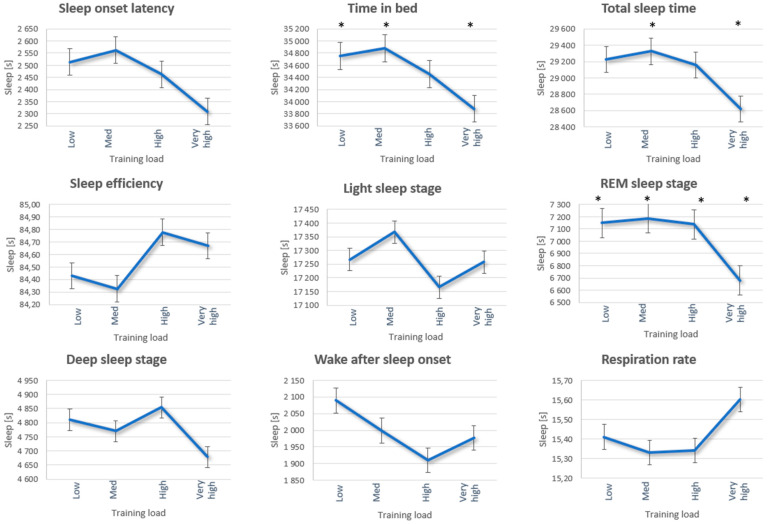
Sleep variation data during low to very high training loads determined by TDI (total distance). * Represents the significant differences.

A multivariate analysis of variance (MANOVA) investigating the aspects between different training loads and sleep variables showed a significant main effect: Pillai’s Trace = 0.023 F(27.6069) = 0.023, *p* = 0.008, Partial Eta Squared = 0.010. Post hoc analysis revealed a decrease of 871.68 s in TIB after a very high training load compared to a low training load (95% [−82.33, −1661.03], *p* = 0.021), and a decrease of 1000.65 s compared to a medium training load (95% [−214.81, −1786.50] *p* = 0.005). The results showed a mean decrease of 707.96 s in TST during a very high training load compared to a medium training load (95% [−12.28, −1403.64], *p* = 0.044). REM sleep was reduced by 468.68 s during a very high training load compared to a low training load (95% [−142.77, −796.60], *p* < 0.001), and was reduced by 507.07 s compared to a medium training load (95% [−181.61, −832.54] *p* < 0.001), and compared to a high training load REM was reduced by 458.49 s (95% [−134.13, −788.86] *p* = 0.001) (see Table 3). None of the other sleep variables were associated with variations in different training loads; see Table 4 or Figure 2 for full reports of these results.

Table 2 and Table 3 show that the football players spent 16 min less TIB when the training loads were very high compared to medium training loads, and 15 min less compared to low training loads. TST was 12 min shorter after very high training loads compared to medium training loads. REM sleep is 7 min shorter after very high training loads compared to high loads, 9 min shorter after very high training loads compared to medium loads, and 8 min shorter compared to low training loads.

## 4. Discussion

The current study aimed to investigate the relationships between different objective training loads classified as low, medium, high, and very high, and sleep variations in female elite football players over one full competitive season.

The main finding was that the players’ sleep duration remained relatively stable until it was disrupted by an excessive training load. It was anticipated that increased training loads would augment the players’ sleep duration; however, an excessively high training load was associated with disturbed sleep patterns.

The results in the current study confirmed the second hypothesis, that the players’ sleep was significantly disrupted (Table 3 and Figure 2) at very high training loads (TIB, TST, REM). This finding raises questions regarding whether the disruption in sleep is due to a lack of structure or awareness in the players’ sleep hygiene practices, or if there are physiological factors affecting their sleep.

### 4.1. Very High Training Load Disrupts Female Football Players’ Sleep

The primary function of sleep is to ensure adequate recovery; therefore, it is presumed that a higher training load would be associated with longer sleep duration, as an increased training load elevates the need for recovery [13]. The current study did not find a positive association between sleep and increased training loads. However, very high training loads were associated with shorter TIB, TST, and REM, which may dictate the opposite of adequate recovery in female football players. As illustrated in Figure 2, there is an inverse relationship between training loads and sleep duration, contrary to the desired outcome. It is concerning that players obtain less TIB and TST when training loads are very high, given the critical importance of sleep in the recovery phase for athletes. [6].

The sleep data categorized under very high training loads likely correspond to nights following match days, as players’ physical exertion peaks during these conditions. This could provide an explanation for the observed findings [7]. Late kick-off hours, bright lights, and traveling make players prone to later bedtimes, as well as post-match formalities, interviews, and/or physical treatment [5]. High caffeine intake with the intended purpose of enhancing performance could also influence the players’ homeostatic and circadian regulations of sleep [5]. After high-intensity or high training volume, elevated core temperatures and increased stress hormone secretion are normal and may disrupt the thermos-physiological cascade to initiate sleep. Elevated core temperature and persistent high heart rate at bedtime following exercise might delay tiredness by delayed selective vasodilation of distal skin regions that promote sleepiness and sleep onset [20]. The coordination of the circadian core temperature rhythm is important for effective sleep, and a lower body temperature is necessary to initiate sleep [21]. Football players experiencing delayed sleep onset may postpone their bedtime, which could account for the observed reductions in TIB and TST during periods of very high training loads. Moreover, TIB is influenced by players’ sleep hygiene practices; reduced TIB may result from a lack of awareness, leading players to choose later bedtimes or wake up too early. Consequently, this disruption in sleep raises concerns about its disproportionate impact on the players’ REM sleep.

The reduced REM sleep might be explained by the physiological recovery processes after the physical efforts from very high training loads. Intense physical exertions, such as sprinting, accelerations, decelerations, and hard tackles often lead to muscle soreness and inflammatory processes [22]. Cortisol is anti-inflammatory and an immune system regulator and REM sleep is found to be associated to the hypothalamus response to physical exertion [23]. The reduced REM sleep observed in the current study after very high training loads may face challenges in regulating the inflammatory process, resulting in an inadequate recovery process since there is a bidirectional association between REM sleep and perceived fatigue, whereas increased REM sleep is associated with reduced perceived fatigue, indicating football players feeling less fatigued and more recovered. On the contrary, a reduced REM phase is associated with increased perceived fatigue, indicating that the players may need more recovery [14]. It is possible that football players may require more sleep to achieve complete recovery after experiencing very high loads, a phenomenon not fully captured by the findings of the present study.

The reduced REM was likely a consequence of shorter TST, whereas the other sleep stages were not influenced. REM sleep predominates during the second half of the night and the REM cycles become longer as the night progresses [24]. This could display a too-early awakening before the players have reached a full sleep restoration since cortisol typically follows a circadian rhythm that peaks early in the morning as REM sleep predominates. Similarly, the growth hormone follows a circadian rhythm but peaks early in the night when DSL predominates [25].

No associations were observed between varying training loads and both DSL and LSL, suggesting that reductions in sleep predominantly affect REM sleep disproportionately compared to other sleep stages. Growth hormone secretion is associated with DSL which has a restorative function on muscle physiology [26]. The reduced REM sleep that was found after very hard loads could also be explained by the growth hormone secretion mediated during DSL [26]. Growth hormones have several functions such as muscle turnover, bone growth, and metabolism regulation, and typically follow a circadian rhythm regulated by several factors such as exercise, nutrition, and sleep. The exact mechanisms for increased growth hormone secretion following exercise are still unknown but may be a combination of the type of exercise, intensity, duration, and frequency [27]. It has been shown that growth hormone secretion is closely associated with elevated blood lactate beyond the anaerobic threshold or resistance training [25]. Consequently, disrupted sleep may result from the recuperation of cells and tissues that occurs during non-REM sleep following an excessively high training load which might lead to deprioritized REM sleep.

Football players aiming to perform daily should optimally be aware of the potential consequences of suboptimal sleep, as disrupted sleep patterns may signal insufficient recovery [5]. Athletes with less variability in sleep have greater sleep efficiency (SE) and fewer sleep variations [28]. The players in the present study struggled with disrupted sleep and less TST and TIB after very high training and match loads. Awareness of sufficient TIB, TST, and consistent sleep onset and offset might add small improvements to football players’ sleep and recovery process, which may lead to positive outcomes in their performance.

### 4.2. Limitations and Strengths

Several limitations should be considered in the interpretation of the current study’s findings. Firstly, a larger sample size would be advantageous. The relatively small number of participants may affect the statistical power and the detection of small effect sizes within the analysis. Secondly, factors such as afternoon napping and the loss of sleep data could potentially impact the results. Thirdly, this study did not incorporate the subjective monitoring of the players’ training loads and sleep. The inclusion of subjective reports could have enhanced the coherence between the football players’ training loads and sleep patterns. This study has several strengths as well. Firstly, the data collection lasted for 9 months, resulting in a substantial amount of datapoints. Measuring sleep over an extended period provides a more accurate representation of the participants’ true sleep characteristics, given the common occurrence of nightly variability. Secondly, the participants are professional female football players from a Norwegian club competing at the highest level. Thirdly, to the best of the authors’ knowledge, this study is the first to explore the associations between training loads and sleep variability among female football players.

### 4.3. Perspectives

The results from the current study show that sleep monitoring could be of importance to optimizing player recovery, which could be beneficial for coaches and athletes in the optimalization of performance development.

The precise functions of sleep and its optimal patterns following different training loads are not yet fully understood. Future research should incorporate both the objective and subjective monitoring of sleep, as well as the quantification of both on-field and off-field training loads, to comprehensively assess how the total workload affects sleep [14]. The complexity of sleep and recovery emphasizes the importance of further research to investigate the potential factors influencing football player’s sleep.

## 5. Conclusions

The present study sheds light on the relationship between the training loads of female football players and fluctuations in their sleep patterns. It was observed that very high training loads were linked to a decrease in TIB compared to low and medium training loads, as well as a reduction in TST compared to medium training loads. Additionally, very high training loads were associated with a diminished duration of REM sleep compared to low, medium, and high training loads. Although the players’ sleep remained consistent following low, medium, and high training loads, it was notably disrupted following very high training loads. The reduced TIB and TST could be associated with a lack of structure or awareness of players’ sleep hygiene. Results of reduced sleep exert a disproportionate impact on REM sleep, which might be a reason for the recuperation of tissues and cells mediated during non-REM sleep. Moreover, players may experience premature awakenings and require additional sleep for comprehensive restoration. It is disconcerting that despite the crucial role of sleep in their recovery processes, there is a lack of improvement in their sleep patterns. Recommending strategies to enhance players’ sleep hygiene, such as extending TIB, could facilitate longer TST and enhance overall recovery.

## Figures and Tables

**Table 1:** Complete list of sleep variables derived from the Somnofy sleep monitor.

Sleep Variable	Abbreviation	Unit	Description
Sleep onset latency	SOL	s	The time it takes to fall asleep after the participant intends to sleep
Time in bed	TIB	s	Total time spent in bed, from bedtime to get up, including sleep and awake
Total sleep time	TST	s	Total sleep time from sleep onset to waking up
Sleep efficiency	SEF	%	The percentage of time spent asleep from SON to SOF
Light sleep	LS	s	Total time in light sleep
Rapid eye movement sleep	REM	s	Total time in REM sleep
Deep sleep	DSL	s	Total time in deep sleep
Wake after sleep onset	WSO	s	Time awake after sleep onset
Respiration rate	RPR	N	Number of respiratory ventilations in 1 min in non-REM sleep

**Table 2:** Descriptive statistics for the sleep variables and TDI grouped into different training loads in 21 female soccer players. Sleep variables are presented in hours and minutes (hh:mm).

Mean (±Standard Deviation)					
	Training Load Based on TDI				
Sleep Variable	Low	Medium	High	Very High	Total
SOL	00:41	00:42	00:41	00:38	00:41
	(±00:32)	(±00:32)	(±00:30)	(±00:27)	(±00:30)
TIB	09:39	09:41	09:34	09:24	09:34
	(±01:19)	(±01:15)	(±01:16)	(±01:24)	(±01:19)
TST	08:07	08:08	08:05	07:56	08:04
	(±01:06)	(±01:06)	(±01:10)	(±01:15)	(±01:10)
SEF	84.43	84.32	84.78	84.67	84.55
	(±7.42)	(±7.95)	(±7.45)	(±7.52)	(±7.59)
LSL	04:47	04:49	04:46	04:47	04:47
	(±00:51)	(±00:51)	(±00:51)	(±00:56)	(±00:52)
REM	01:59	02:00	01:58	01:51	01:57
	(±00:33)	(±00:31)	(±00:31)	(±00:34)	(±00:32)
DSL	01:20	01:19	01:20	01:17	01:19
	(±00:22)	(±00:22)	(±00:23)	(±00:22)	(±00:22)
WSO	00:34	00:33	00:31	00:32	00:33
	(±00:30)	(±00:28)	(±00:31)	(±00:30)	(±00:30)
RPR	15.41	15.33	15.34	15.60	15.42
	(±1.95)	(±1.89)	(±1.91)	(±1.93)	(±1.92)
TDI (m)	2622.04	3931.8	5163.62	8968.58	5173
	(±744.093)	(±498.478)	(±897.484)	(±176.896)	(±2225)
N	501	510	517	505	2033

SOL, sleep onset latency; TIB, time in bed; TST, total sleep time; SEF, sleep efficiency; LSL, light sleep; REM, rapid eye movement sleep; DSL, deep sleep; WSO, wake after sleep onset; RPR, NREM respiration rate per minute; TDI, total distance.

**Table 3:** Test of between-subjects effects and multiple comparisons investigating the effect of different training loads on sleep variables in 21 female soccer players. The significant variables are presented in this table.

ANOVA within Each Variable				Multiple Comparisons						
Dependent Variable	DF	F	*p*-Value	Training Load		Mean Difference (I–J)	Std. Error	Sig.	95% Confidence Interval	
									Lower Bound	Upper Bound
TIB	3	4.464	0.004 *	Very high	Low	−871.68 *	298.900	0.021	−1661.03	−82.33
					Med	−1000.65 *	297.573	0.005	−1786.50	−214.81
					High	−569.44	296.569	0.330	−1352.64	213.75
TST	3	2.895	0.034 *	Very high	Low	−610.32	264.606	0.127	−1309.11	88.46
					Med	−707.96 *	263.432	0.044	−1403.64	−12.28
					High	−541.66	262.543	0.235	−1234.99	151.68
REM	3	7.570	0.001 *	Very high	Low	−469.68 *	123.792	0.001	−796.60	−142.77
					Med	−507.07 *	123.242	0.000	−832.54	−181.61
					High	−458.49 *	122.827	0.001	−782.86	−134.13

Based on observed means. The error term is Mean Square(Error) = 3.709. * The mean difference is significant at the 0.05 level. Note: DF, Degrees of Freedom; F, F-value; TIB, time in bed; TST, total sleep time; REM, rapid eye movement sleep.

**Table 4:** Test of between-subjects effects and multiple comparisons investigating the effect of different training loads on sleep variables in 21 female soccer players.

ANOVA within Each Variable				Multiple Comparisons						
Dependent Variable	DF	F	*p*-Value	Training Load		Mean Difference (I–J)	Std. Error	Sig.	95% Confidence Interval	
									Lower Bound	Upper Bound
SOL	3	1.781	0.149	Very high	Low	−204.23	116.519	0.479	−511.94	103.48
					Med	−253.08	116.001	0.175	−559.43	53.26
					High	−154.04	115.610	1.000	−459.35	151.27
TIB	3	4.464	0.004 *	Very high	Low	−871.68 *	298.900	0.021	−1661.03	−82.33
					Med	−1000.65 *	297.573	0.005	−1786.50	−214.81
					High	−569.44	296.569	0.330	−1352.64	213.75
TST	3	2.895	0.034 *	Very high	Low	−610.32	264.606	0.127	−1309.11	88.46
					Med	−707.96 *	263.432	0.044	−1403.64	−12.28
					High	−541.66	262.543	0.235	−1234.99	151.68
SEF	3	0.387	0.762	Very high	Low	0.2396	0.47887	1.000	−1.0250	1.5042
					Med	0.3431	0.47675	1.000	−0.9160	1.6021
					High	−0.1093	0.47514	1.000	−1.3641	1.1454
LSL	3	0.348	0.790	Very high	Low	−9.23	199.551	1.000	−536.21	517.76
					Med	−109.71	198.665	1.000	−634.35	414.93
					High	91.93	197.995	1.000	−430.94	614.80
REM	3	7.570	0.001 *	Very high	Low	−469.68 *	123.792	0.001	−796.60	−142.77
					Med	−507.07 *	123.242	0.000	−832.54	−181.61
					High	−458.49 *	122.827	0.001	−782.86	−134.13
DSL	3	1.523	0.207	Very high	Low	−131.40	85.986	0.760	−358.47	95.68
					Med	−91.17	85.604	1.000	−317.23	134.90
					High	−175.04	85.315	0.242	−400.35	50.26
WSO	3	0.855	0.464	Very high	Low	−111.56	113.681	1.000	−411.78	188.66
					Med	−21.39	113.177	1.000	−320.27	277.49
					High	67.67	112.795	1.000	−230.21	365.54
RPR	3	2.145	0.093 *	Very high	Low	0.1910	0.12143	0.696	−0.1297	0.5117
					Med	0.2697	0.12090	0.155	−0.0496	0.5889
					High	0.2604	0.12049	0.185	−0.0578	0.5786

The error term is Mean Square(Error) = 3.709. * The mean difference is significant at the 0.05 level. Note: SOL, sleep onset latency; TIB, time in bed; TST, total sleep time; SEF, sleep efficiency; LSL, light sleep; REM, rapid eye movement sleep; DSL, deep sleep; WSO, wake after sleep onset; RPR, NREM respiration rate per minute.

## Data Availability

The data supporting the conclusions of this article will be made available by the authors due to privacy and ethical restrictions.
